# Phytochemical Investigation, Antimicrobial, Antioxidant and Anticancer Activities of *Acer cappadocicum* Gled

**DOI:** 10.3390/life11070656

**Published:** 2021-07-05

**Authors:** Farzana Kausar, Muhammad-Awais Farooqi, Hafiz-Muhammad-Umer Farooqi, Abdul-Rahim-Chethikkattuveli Salih, Atif-Ali-Khan Khalil, Chul-woong Kang, Mohamed H. Mahmoud, Gaber-El-Saber Batiha, Kyung-hyun Choi, Abdul-Samad Mumtaz

**Affiliations:** 1 Department of Plant Sciences, Quaid-i-Azam University, Islamabad 45320, Pakistan; kausarfarzana4915@gmail.com; 2 Department of Mechatronics Engineering, Jeju National University, Jeju-si 63243, Korea; awaisfarooqi@stu.jejunu.ac.kr (M.-A.F.); umerfarooqi@jejunu.ac.kr (H.-M.-U.F.); abdul.rahim@jejunu.ac.kr (A.-R.-C.S.); cukang@jejunu.ac.kr (C.-w.K.); 3 National Control Laboratory of Biologicals, Drug Regulatory Authority of Pakistan, Islamabad 44090, Pakistan; 4 Department of Biological Sciences, National University of Medical Sciences, Rawalpindi 46000, Pakistan; atif.ali@numspak.edu.pk; 5 Department of Biochemistry, College of Science, King Saud University, Riyadh 11451, Saudi Arabia; mmahmoud2@ksu.edu.sa; 6 Department of Pharmacology and Therapeutics, Faculty of Veterinary Medicine, Damanhour University, Damanhour 22511, Egypt; dr_gaber_batiha@vetmed.dmu.edu.eg

**Keywords:** *Acer cappadocicum*, antibacterial activity, antioxidant activity, cytotoxicity

## Abstract

The appearance of novel microbial resistance, diverse cancer ailment and several other morbidities such as appetite loss, hair loss, anemia, cell damage, etc., are among most critical situation that keeps the phytochemical quest on. Thus, this study characterized the antimicrobial, antioxidant, and anticancer potentials of a rarely accessed *Acer cappadocicum* gled (AC) population thriving in a remote Palas Valley in northern Pakistan. Leaf extracts of the plant were prepared in organic solvents with different polarities through maceration. Extracts were subjected to antimicrobial, antioxidant, and anticancer activities using agar well, DPPH and cell viability assays. *A. cappadocicum* methanolic extract (ACM) significantly inhibited bacterial growth, followed by *n*-butanolic extract (ACB) with the second-highest bacterial inhibition. Similar activity was observed against mycelial growth inhibition in plant-fungal pathogen by ACM and ACB. However, human pathogenic fungi did not affect much by extracts. In antioxidant assessment, the chloroform extract (ACC) showed strong scavenging activity and in cytotoxic evaluation, extracts restricted growth proliferation in cancer cells. The inhibitory evidence of extracts, potent scavenging ability, and low cell viability of human-derived cell lines supports the antimicrobial, antioxidant and anticancerous potential of *A. cappadocicum.* It advances our quest for natural product research.

## 1. Introduction

Infectious diseases caused by different bacterial species, e.g., *Staphylococcus aureus*, *Pseudomonas aeruginosa*, *Bacillus subtilis* and *Proteus vulgaris*, pose major public health concerns worldwide. The antibacterial drugs meant to resist infectious agents lose their effectiveness due to the accumulating mutations in bacterial species [1]. Thus antibiotic resistance capability in bacteria eventually becomes very serious in treating pathogenic strains [2,3]. Other morbidities, for instance, cancer, have also been a major cause of death and pose a constant threat worldwide, which is characterized by dysregulation of signalling pathways at multiple steps during cell division. The cells in the human body undergo uncontrolled division resulting in tumor formation of malignant cells with metastatic potential. Recent treatments include chemoprevention, radiation exposure and synthetic medicines [4].

Furthermore, several human ailments are due to the occurrence of free radicals in the human body. These unstable free radicals tend to attain stable form by donating their free electrons to DNA, protein, amino acids, etc., causing extraordinary changes at cellular levels [5,6]. Occasionally, to overcome the deficiency of endogenous antioxidants, it is necessary to rely on herbal and dietary sources of antioxidants. Phytochemicals, such as carotenoids, tannins, polyphenols and phenolics, act as natural radical scavengers and organic sources for antioxidants [7].

Due to antibiotic resistance and toxicity issues of antibacterial agents, it is an unmet need to discover new and novel sources to combat bacterial diseases. The lack of effectiveness in treatment and the risk of potential side effects pushed researchers to find advanced chemoprotective solutions stipulating from plant extracts as natural compounds or derived synthetically from natural prototypes [3]. Many cultivated and wild plant species have been investigated for their inhibiting potential of free radicals. Several studies have successfully led to the drug discovery processes from natural sources, thus increasing interest in various “good food for health” and finding diversity in Phyto-antioxidant sources. Plant metabolites responsible for high antioxidant potential, mostly flavonoid and phenolics, can be further characterized by biochemical tests and spectroscopic methods [8,9,10]. Recently, plants are not only used for their nutritional value but also for treating many health problems. Much consideration has been given to plant extracts possessing different chemical constituents to inhibit bacterial infections. According to Murugan et al. [11], about 80% of the population in developing countries rely on plants for therapeutic purposes. 

Plants are a natural source of secondary metabolites, e.g., saponins, tannins, alkaloids, phenolic and flavonoids, etc. These metabolites rich in their structural and functional diversity provide various effective bioactive compounds in extracts that prove efficacious against bacterial infections and cancer development. Moreover, these are less toxic to healthy human cells [12,13]. The genus *Acer* (a member of the family Aceraceae) is comprised of 129 species globally. Predominantly trees or shrubs, this plant family is famous for traditional uses such as anti-rheumatic, anti-hepatic, against eye disorders, treating bruises and a detoxifier. Members of Genus Acer are comprised of a variety of molecules with diverse functional groups. There have been extensive phytochemical studies on extracts of various Acer species around the world. However, its biological activities remain less discovered till today. Numerous *Acer* species have been investigated for their biological and pharmacological potentials [14]. *Acer cappadocicum* (Aceraceae) is a tall tree native to the western Himalayas of Pakistan. Taxonomically synonymized as *Acer cultratrum* wall., *Acer laetum* C.A. Meyer and *Acer pictium*, is locally known as kilpattar or Chinaranga in Pallas valley, Kohistan, KP, Pakistan [2,3]. Traditionally, *A. cappadocicum* improves blood circulation and relieves pain due to injury or rheumatisms [15]. In the Kohistan region, *Acer cappadocicum* is an endemic species, and its roots are locally claimed to heal animal foot disease. Hitherto, this species remains undocumented for biological and pharmacognostic aspects. Therefore, the main objective of the current study was to investigate the antimicrobial effect on human pathogenic strains, the antioxidant potential against free radicals, the anticancerous potential on liver, breast, lung and gut cell lines and the phytochemical aspects of various *A. cappadocicum* extracts.

## 2. Materials and Methods

### 2.1. Collection and Preparation of Plant Material

The branches of *A. cappadocicum* with green leaves were collected from Pallas valley, District Kohistan, Khyber Pakhtunkhwa, Pakistan. The collected plants were authentically identified with the help of a senior plant taxonomist and submitted to the herbarium Quaid-i-Azam University, Islamabad, Pakistan.

Plant extracts were prepared through maceration following a previously reported method [16]. Dry leaves and branches were pulverized mechanically in a willy mill with mesh size 60, soaked in a solvent (20 g/200 mL) and placed over a shaker for 72 h. The material was then filtered through Whatmann No.1 filter paper and was evaporated at room temperature under shade. The resulting crude extract was then suspended in water and partitioned with *n*-hexane, ethyl acetate (EtOAc), chloroform and *n*-butanol (*n*-BuOH). The concentrated extracts were stored at 4 °C till further use. 

### 2.2. Preliminary Phytochemical Analysis

The crude extract was subjected to a qualitative chemical test for the detection of phytochemicals such as flavonoids using a sodium hydroxide and magnesium ribbon test, alkaloids using the Mayer’s reagent test, the Keller–Killani test for glycosides, for saponins, a froth and emulsion test, for terpenoids (phytosteroids), a chloroform and sulphuric acid test, for tannins, a ferric-chloride and alkaline reagent test, and for proteins, xanthoproteic and Biuret tests, as per reported methods [17,18].

### 2.3. Antimicrobial Assays

Antimicrobial potential of plant extracts was evaluated using five pathogenic bacterial and four fungal strains. For antibacterial activity, the bacterial strains selected were: *Kelabsiella pneumoniae* (82431), an opportunistic pathogen with a history of chronic pulmonary diseases, *Escherichia coli* (52321), *Bacilus subtilis*, *Salmonella enterica* and *Acinetobacter baumannii*. These strains were obtained from the Department of Microbiology, Quaid-i-Azam University Islamabad. The agar well diffusion method was used for evaluating the antimicrobial potential of plant extracts [19,20]. Nutrient agar was prepared for bacteria according to the manufacturer’s instructions. Immediately after autoclaving, the media was allowed to cool at 45–50 °C in the water bath. The freshly prepared cooled media was poured (approximately 4mm depth) into flat bottom Petri dishes (90 mm in diameter). About 0.2 mL of test inoculum of bacteria was spread uniformly on the surface of solidified agar media using a sterile L-shaped glass rod. Four equidistant wells with a 5 mm diameter were made and 4 mm in depth on the agar using a sterile cork borer. Two more wells for positive and negative control at the middle of the agar plate were also made. About 25 μL of plant extracts (in a concentration of 4 mg/mL) and the controls were added into the wells. The positive and negative control wells received Kinamycin and DMSO, respectively. The wells were labelled carefully to correspond to the crude extracts and controls. The plates were stored at 4 °C for at least six hours to allow diffusion of extracts into agar, arresting the growth of test microbes. The plates were then incubated for 24 h at 37 °C. The test was carried out in triplicates. Antimicrobial activity was determined by measuring the diameters of the zone of inhibition.

For another study, the agar tube dilution method was used to elicit the extracts’ effects on the phytopathogen reported by Choudary et al. 1995. Three strains were used, *Fusarium fujikuroi*, *Rhizopus oryzae* and *Pythium ultimum*, which are plant pathogens causing disease in various economical plants. The results were determined as described previously [21].

$$\mathrm{Percentage}\;\mathrm{inhibition}\;\mathrm{of}\;\mathrm{fungal}\;\mathrm{growth}=\frac{100-\;\mathrm{linear}\;\mathrm{growth}\;\mathrm{in}\;\mathrm{test}\;\mathrm{tube}\;}{\mathrm{linear}\;\mathrm{growth}\;\mathrm{in}\;\mathrm{control}}\times 100$$

### 2.4. DPPH Free Radical Scavenging Assay

A stock solution of 40,000 μg/mL was prepared by dissolving 40 mg of test plant extract in 1 mL of DMSO. Further working dilutions of concentrations 100 μg/mL, 200 μg/mL, 300 μg/mL, 400 μg/mL, 500 μg/mL, 600 μg/mL, 700 μg/mL, 800 μg/mL, 900 μg/mL and 1000 μg/mL were prepared by serial dilution method. DPPH solution (0.1 mM) was prepared by dissolving 0.975 mg DPPH in 25 mL of methanol. Then it was placed on a magnetic stirrer for 30 min in the dark. For the assay, 190 μL of freshly prepared DPPH solution and 10 μL of each sample was mixed in a 96-Well Microtiter Microplate. The negative and positive controls received Ascorbic acid and DMSO, respectively. The reaction plate wrapped in an aluminum foil was incubated at 37 °C for 30 min. The absorbance of the test samples was measured at 517 nm. The percentage scavenging activity was calculated following [22,23], as described below:

$$\mathrm{Scavenging}\;\mathrm{activity}\%\;=\frac{\mathrm{Abs}\;\mathrm{of}\;\mathrm{control}\;–\;\mathrm{Abs}\;\mathrm{of}\;\mathrm{sample}\;}{\mathrm{Abs}\;\mathrm{of}\;\mathrm{control}\;}\times 100$$

### 2.5. Anticancer Assays

The human-derived cell lines were purchased from Korea Cell line bank, South Korea. These cell lines were grown in Roswell Park Memorial Institute (RPMI) 1640 cell culture media. In contrast, normal cells (primary human cells) were cultured with their respective specific cell culture media. Cell culture media (Thermofisher, Grand Island, NY, USA) were supplemented with 10% fetal bovine serum (FBS) and 1% *v*/*v* Penicillin /Streptomycin antibiotic solution for cell cultures (Thermofisher, Grand Island, NY, USA). The cells were kept in a humidified incubator with 5% CO_2_ at 37 °C. The cytotoxic effect of plant extracts was investigated by MTS assay using Tetrazolium dye (Thermofisher, Grand Island, NY, USA) reduction to formazan deep purple soluble crystals through the metabolic activity of cells. In addition, cell viability was assessed as a function of absorbance at 490 nm.

First, we revived the preincubated cells. The cells were seeded in 96 well-microtiter plates at a concentration of 2000 cells per well/500 μL, followed by overnight incubation in the humidified environment with 5% CO_2_ at 37 °C. After that, cells were treated with different concentrations (1 mg/mL to 0.1 ng/mL) of plant extracts prepared by serial dilutions for 48 h in the environment of 5% CO_2_ at 37 °C. The negative control contained cells and media only without test extracts. The positive control had cells, media and tested samples. After 48 h of incubation, the cells were washed with PBS/Dulbecco’s phosphate buffer saline (DBPS) and followed by addition by MTS reagent 25 μL solution. The plate was incubated at 5% CO_2_ and 37 °C for 30 min. Absorbance was measured at 490 nm using a multimode microplate reader (SpectraMax i3x). The cell viability was estimated by (x − y)/z − y) × 100%, where x was the absorbance of sample, y was the absorbance of blank, and z is the absorbance of control. The IC_50_ value was calculated by obtaining a linear equation from a plot between the percentages of cell viability vs concentrations. A ‘P’ value *p* < 0.05 was considered as significant [24,25,26].

## 3. Results and Discussion

### 3.1. Phytochemical Screening

The results of the phytochemical screening of the extracts are summarized in Table 1. The members of Aceraceae are characterized by the presence of flavonoids and tannins, etc. [14]. The alkaloids, glycosides and proteins were confirmed in all extracts. Tannins were present only in methanolic and butanoic extracts. Steroids were found in ACM and phytosteriods in ACB and ACE.

### 3.2. Antimicrobial Assay 

According to the current study, antibacterial activities of methanolic extract at a concentration of 40 mg/mL showed a maximum inhibition zone of diameters of 16.5 ± 1.0 mm against *B. subtilus*, *E. coli* and *K. pneumonia* and 15.0 ± 1.0 mm and 11 ± 2 mm against *S. entrica* and *A. baunanni*, respectively (Table 2). The *n*-butanolic extracts were not significantly different from methanolic extracts with inhibition zone diameters with the mean value of 15.0 ± 1.0 mm, 14.5 ± 1.0 mm and 15.0 ± 1.0 mm against *B. subtilis*, *E. coli* and *K. pneumonia*, respectively. Furthermore, the zone of inhibition exhibited by *n*-butanolic extracts against *S. entrica* is 11.5 ± 1.0 mm and 16.0 ± 1.0 mm, which is the highest value against *A. buamanni* among all extracts [20,27].

Plant extracts were also tested for pathogenic fungal strains mentioned as *A. falvus*, *A. niger* and *Pythium* sp. The extract showed no characteristic mycelial inhibition against fungal species. The percentage of inhibition of mycelial growth of fungal strains *F. fujikuroi*, *R. oryzae* and *P. ultimum* by plant extracts shown in Table 3. *F. fujikuroi*, a plant pathogen, causes bakanae disease in rice plants resulting in extraordinary elongation of diseased plants, eventually resulting in plant death [28]. *R. oryzae* is an opportunistic human pathogen causing Mucormycosis and pulmonary infection in immunocompromised patients [29] and *P. ultimum,* a plant pathogen causing damping-off and root rot disease in a variety of economical plants [30]. All extracts showed moderate activity, around 40–50%. The lowest inhibition was 34% by chloroform extract against *F. fujikuroi*, and the highest was 56% against *P. ultimum* of *n*-hexane extract [17,31].

### 3.3. Antioxidant Activity

The presence of free radicals in the human body results in various ailments. Antioxidants present in plants have a natural tendency to scavenge free radicals. DPPH is an easy, standardized and economical procedure to investigate the antioxidant potential in phytoextracts using a spectrophotometer [20,21]. IC_50_ value, the half-maximal inhibitory concentration was calculated to determine the 50% inhibition of DPPH radical by plant extracts. It was calculated by linear regression analysis, the lower the value of IC_50,_ the higher the antioxidant ability of plant extracts [21,31]. The results indicate that chloroform extract has the strongest scavenging activity compared to other extracts. The IC_50_ values of all sample extracts are 0.5 23 (ACB), 0.5146 (ACE), 0.5987 (ACM) and 0.5108.

**Figure 1:**
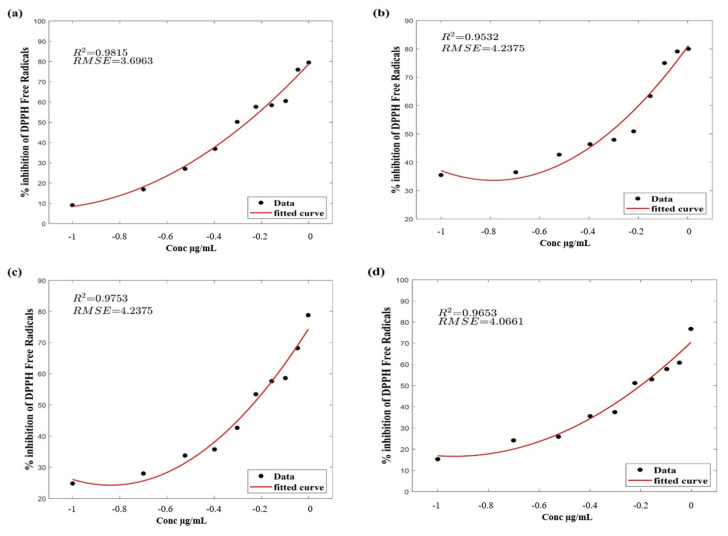

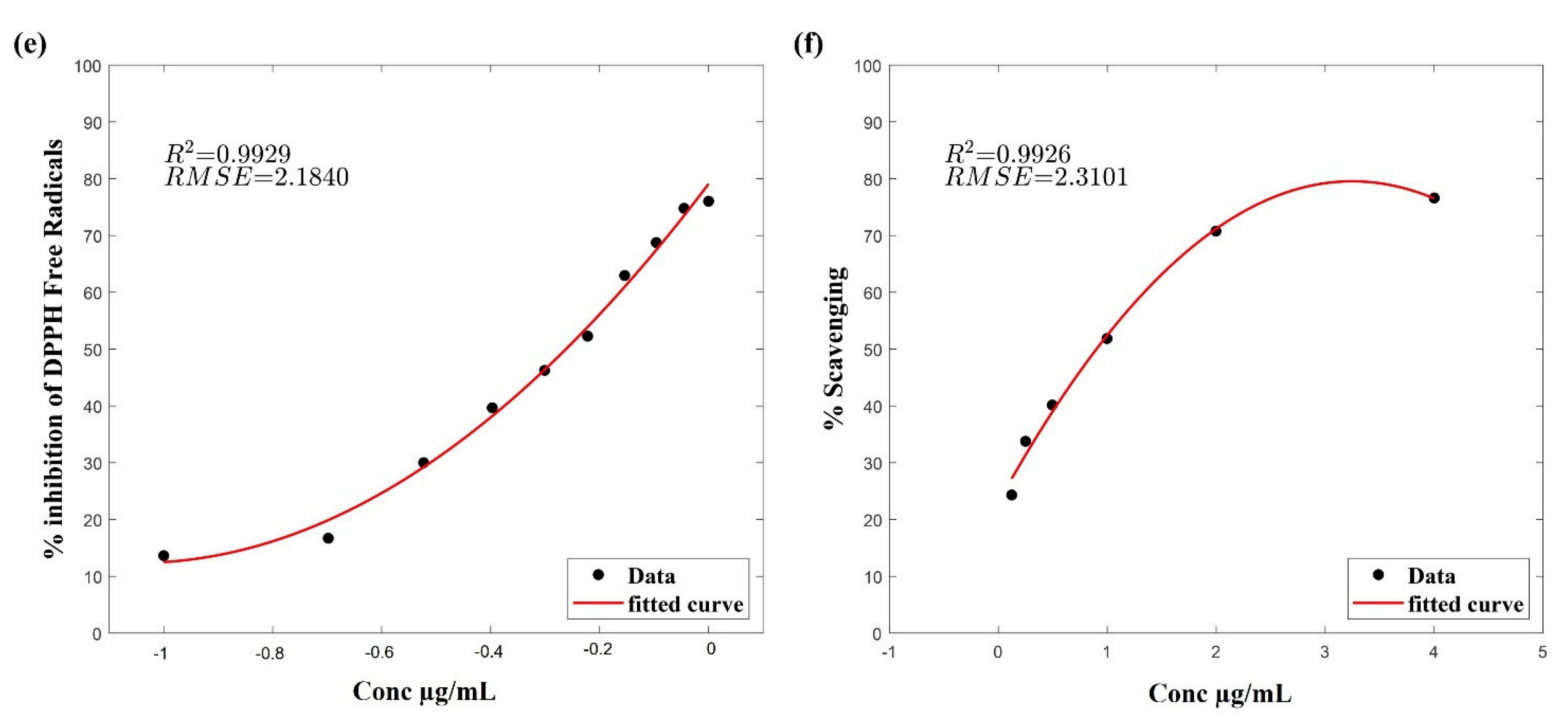
Free radical scavenging of plant extracts by the DPPH method at different concentrations (μg/mL). Here, (**a**): ACB extract, (**b**): ACC extract, (**c**): ACE extract, (**d**): ACM extract, (**e**): ACN extract, (**f**): standard (ascorbic acid).

The results of DPPH radical scavenging (%) at various concentrations are illustrated in Figures (Figure 1a–f). From these figures, it is clear that plant extracts exhibited strong DPPH inhibitory activity. As shown in the figures, the inhibition rate of extracts is in concentration-dependent form and expressed as IC_50_ value (Table 4), which requires concentration of samples necessary for 50% inhibition of DPPH by extracts [32]. At higher concentrations, the activity of extracts was also higher. At a lower concentration (1.0 μg/mL), the extracts exhibited similar activity (Figure 1a,c,e). The ACC (Figure 1b) showed maximum inhibiting of DPPH with similar results of *B. vahili* extract in a study by K. Sowndhararajan and S.C. Kang, 2012 [33]. 

### 3.4. Anticancer Activity

**Figure 2:**
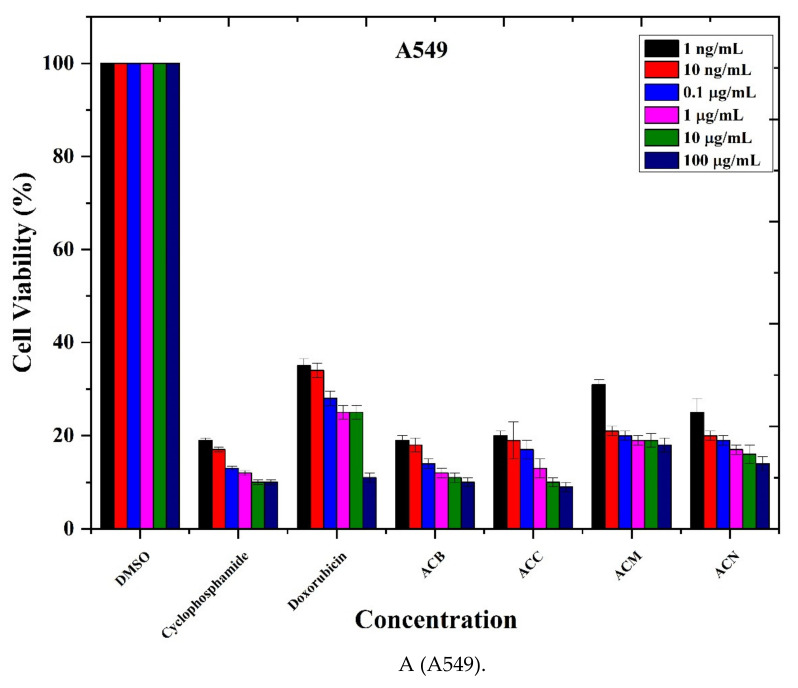

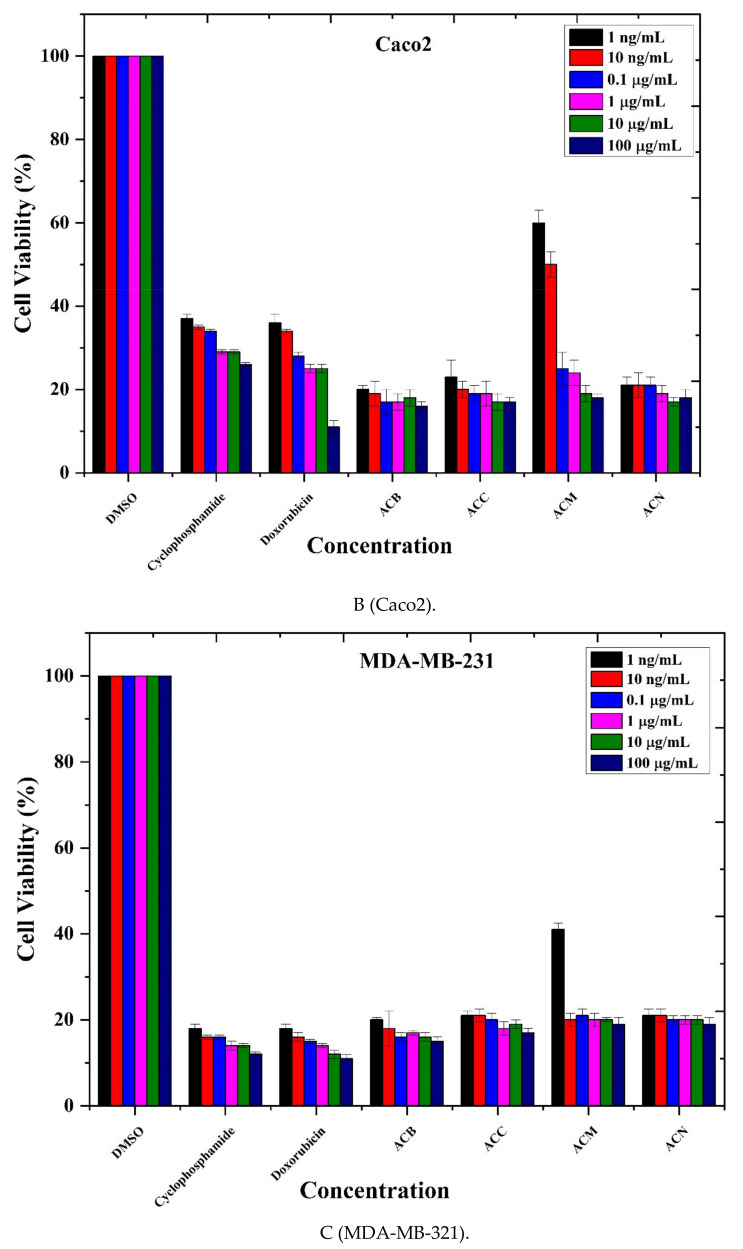

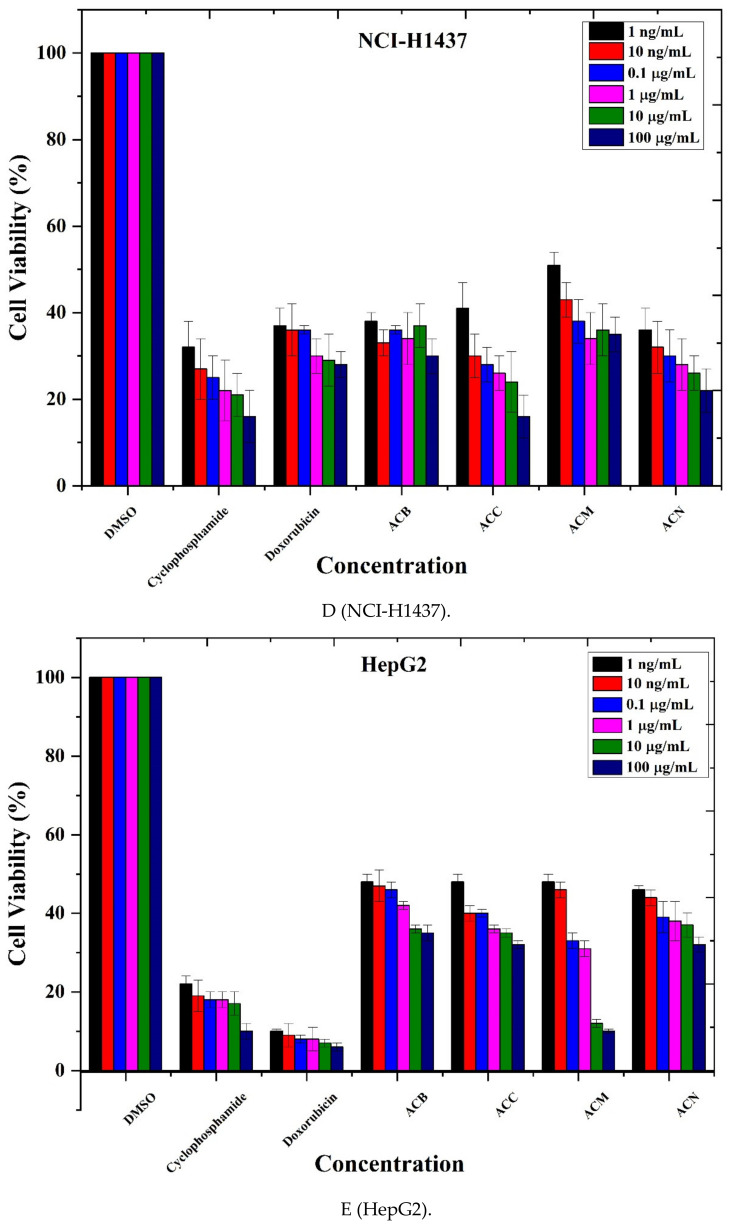

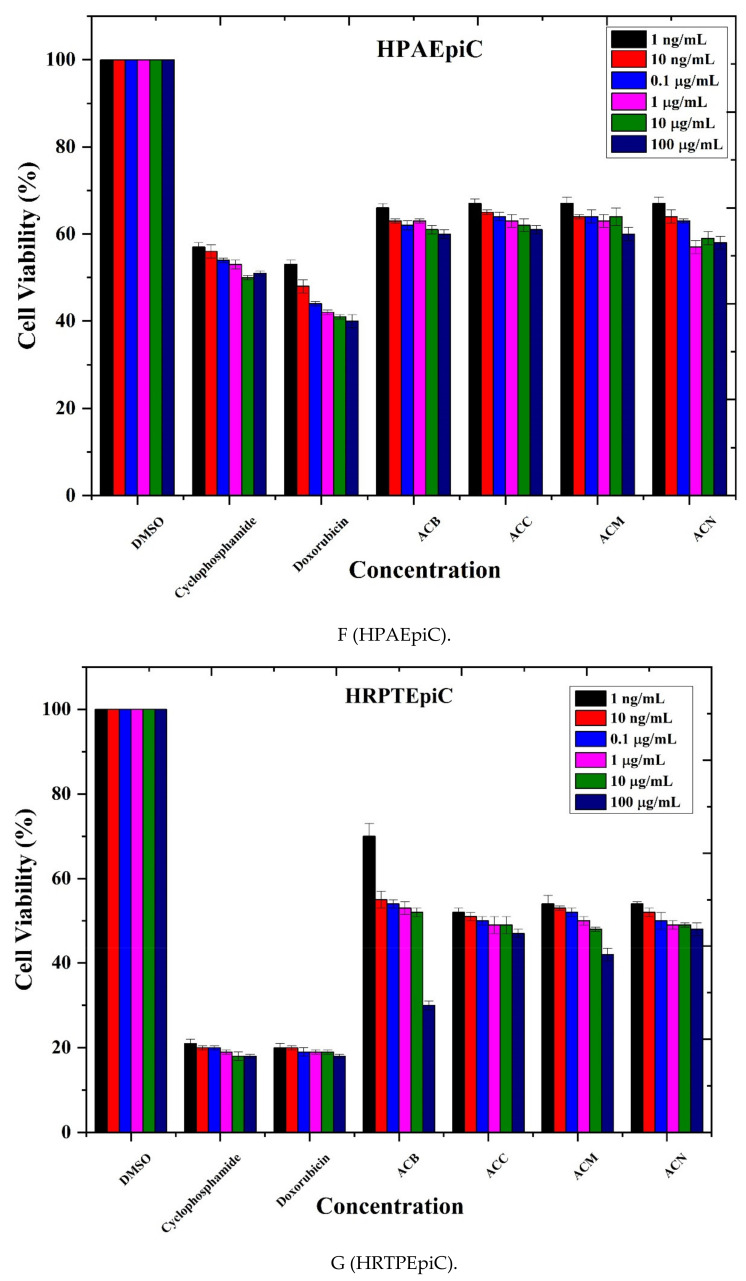
**Cell viability:** MTS assay histograms represent the percentage with respect to control cells (positive control: 20–40% viable cells) after the exposure to 1 ng/mL, 10 ng/mL, 0.1 μg/mL, 1 μg/mL, 10 μg/mL and 100 μg/mL of ACB, ACC, ACM and ACN extracts in Caco 2 cells (**A**), A549 (**B**), MDA MB-321 (**C**), NCI-H1437 (**D**), HepG2 (**E**) cancerous cell lines and HPAEpiC cells (**F**) and HRPTEpiC (**G**) cell lines. Data shows mean ± SE (n = 3).

The anticancer effects of extracts from *A. cappadocicum* on the growth of five different human cancer cell lines named Gut cancer (Caco 2 cells), Lung (A549), Breast (MDA MB-321), Lung cancer (NCI-H1437), liver hepG2 cells line and two normal cell lines named Human pulmonary alveolar epithelial cells (HPAEpiC) and Human renal proximal epithelial cells (HRPTEpiC) from ScienceCell were investigated by MTS assay. MTS upon reduction by cells metabolic activity formed soluble formazon dye and quantified by spectroscopic method [23,24]. The cell survival of *n*-butanolic, chloroform, methanolic and *n*-hexane extracts was in the order of MDA MB-321 > A549 > Caco2 cells without much difference regarding concentrations (Figure 2A–C). The extracts exhibited strong cytotoxic activity with 20–30% cell survival [34,35]. In addition to that, the cell viability effect on NCH-H1437 chloroform extract shows maximum anticancer activity with low cell viability. In Hepg2 cells, the methanolic extract showed the highest activity with the low survival rate of cells (Figure 2D,E) [36].

The plant extracts exhibited significant (*p* < 0.05) cytotoxicity against the cancerous cell, while normal cells (primary lung alveolar and renal primary tubular epithelial cells) appeared less sensitive to plant extracts. Relative much higher cell viabilities for normal cells suggested plant extracts were safe/nontoxic to normal cells [37]. The studies have shown that phenolic-rich extracts of *Acer* species inhibit cell proliferation in tumor cell lines [38]. In another study [39], it is suggested that *Acer tegmentosum* can be a source of antioxidant and anticancer drugs. Despite many chemical studies in maple plants and interests in its constituents, the cytotoxic effect of Acer species and *A cappadocicum* still not documented in the literature. In the following research, four types of extracts were used to perform MTS assay, and the results were expressed the percentage of cell viability. Notably, similar to our study, *Acer* extracts can inhibit the growth of the cells in human cell lines [39]. Plant extracts considerably inhibited cell survival, providing that AC can be a supplementary material for anticancer drug research.

As illustrated by Figure 2, the AC extracts exhibited significant cytotoxicity in cancerous cells in a dose-dependent manner. We screened five different cell lines for inhibition of cancerous activities. Out of five, four cancerous cell lines showed a notable decrease in cell survival rate, implying that extracts are exceptionally active towards cytotoxic effect. This can be attributed to tannins, quinones, steroids, phytosteriods and alkaloids in AC extracts. Moreover, Plant extracts exhibited a significant (*p* < 0.05) cytotoxicity towards lung (A549) and breast (MDA-MB 321) cell lines in a dose-dependent manner compared to other HepG2, HPAEpi and HPRTEpi cell lines (*p* < 0.05), respectively, while the normal cells showed much less sensitivity. The IC_50_ value was calculated to be 5.88, 6.16, 7.11 and 8.97 in A549, caco2, MDA-MB321 and NCI-H1437, respectively (*p* < 0.05), whereas normal cells showed relatively higher IC_50_ values. These results were also compared with two standard commercial anticancer drugs, cyclophosphamide and doxorubicin, for the comparative analysis of cell survival rate shown in Figure 2.

The overall potential of cytotoxic in A549 was 10–20%, followed by MDA-MB321 up to 20% and then caco2 cell >20%. In NCI-H1437, 30–40% cell survival rate was followed by 40–50% in HepG2 cells. HepG2 cells showed less cytotoxicity in all tested cell lines as shown in Table 5. Further to this, the cell survival rate in normal cell lines was also studied. The percentage cell viability was 50–68% in HPAEpic and 50–60% in HPRTEpiC, which showed that extracts are less toxic on healthy cells. These findings suggest that *A. cappadocicum* extracts are exceptionally bioactive against cancerous cell lines with a very low cell survival rate in A549, MDA-MB321 and Caco2 cells. Therefore, the AC plant can be an excellent natural material for anticancer studies in the lung (A549), Breast (MDA-MBA321) and gut (caco2) cancers.

## 4. Conclusions

To the best of our knowledge, this is the first report of AC extracts to determine antibacterial, antioxidant and anticancer properties. The present study showed that AC extracts possess potential antibacterial activity against human pathogens and moderate mycelial inhibition of plant pathogens. In addition, extracts of *A. cappadocicum* have also shown a significant reduction in cancer cell line proliferation. These findings showed that AC is a rich source of bioactive chemicals with significant potential as nutraceuticals and health benefits. These properties can be connected to intrinsic active secondary metabolites found in AC leaves, including alkaloids, glycosides, quinones and tannins. However, more epidemiological work, research investigations and clinical trials are required in order to isolate the pharmacologically potent compounds that can contribute to the observed effects and explain the possible mechanism of actions of the plant and their potential efficacy on various medicinal formulations and severe diseases.

## Figures and Tables

**Table 1:** Phytochemical screening of *A. cappadocicum* extracts.

Constituents	Tests	ACB	ACC	ACE	ACM	ACN
Alkaloids	Mayer’s reagent test	+	+	+	+	+
Tannins	FeCl_3_ test	+	−	−	+	−
	Alkaline reagent test	+	−	−	+	−
Sterol		−	−	−	−	−
Steroids		−	−	−	+	−
Phytosteroids		+	−	+	−	−
Glycosides		+	+	+	+	+
Quinones		+	+	+	+	−
Protein detection	xanthoprotiec test	+	+	+	+	+

Notes: +: Positive test; −: Negative test.

**Table 2:** Antibacterial screening of *A. cappadocicum* extracts against Gram-positive and Gram-negative bacteria.

Isolates	*B. subtilus*	*E. coli*	*K. pneumonia*	*S. entrica*	*A. baumanni*
Extracts	Zone of Inhibition (mm)				
ACB	15	14.5	15	11.5	16
ACC	9.5	10.5	10	0	14
ACE	12	13.5	14.5	12	13
ACM	16.5	16.25	16.5	15	11
ACN	13	11	11.5	0	12

Zone of inhibition (mm) are expressed as the mean, ACB: *n*-butanolic extract, ACC: chloroform extract, ACE: ethylacetate extract, ACM: metholic extract and ACN: *n*-hexane extract of *A. cappadocicum.*

**Table 3:** The percentage of inhibition of mycelial growth of *F. fujikuroi, R. oryzae* and *P. ultimum* by plant extracts.

Extracts	*F. fujikuroi*	*R. oryzae*	*P. ultimum*
ACB	47	47	53
ACC	34	45	53
ACE	50	51	52
ACM	48	52	49
ACN	40	49	56
Positive control	56	79	62

% age mycelial inhibition expressed as mean ± SD (n = 3); lower inhibition = 20–30%, moderate inhibition = 40–50%; high inhibition = 60–80%, Positive control; Terbinafine.

**Table 4:** The IC_50_ value of DPPH radical scavenging activity.

Sample	IC_50_ (μg/mL)
ACB	0.5233
ACC	0.3597
ACE	0.5146
ACM	0.5987
ACN	0.5108
Ascorbic acid	15.67

**Table 5:** The percentage cell viability rate and IC_50_ values of extracts against various cell lines.

	Cell Lines	% Age Cell Viability	IC_50_ Value
Cancer Cell Lines	A549	20%	5.88
	Caco 2	10–20%	6.16
	MDA MB-321	20%	7.11
	NCI-H1437	30–35%	8.97
	HepG2	40–50%	9.11
Normal Cell Lines	HPAEpiC	57–68%	13.44
	HRPTEpiC	50–60%	14.85

## Data Availability

The data supporting this study are available from corresponding author upon reasonable request.
